# Sociocultural and linguistic boundaries influencing intercultural communication between nurses and Moroccan patients in southern Spain: a focused ethnography

**DOI:** 10.1186/1472-6955-12-14

**Published:** 2013-05-24

**Authors:** Fernando J Plaza del Pino, Encarnación Soriano, Gina MA Higginbottom

**Affiliations:** 1 Department of Humanities and Education Science, Universidad de Almeria, Ctra. Sacramento s/n, La Cañada de San Urbano, Building No. 2 (Building C), Office 0.222, 04120, Almería, Spain; 2 Faculty of Nursing, University of Alberta, 3rd Floor Edmonton Clinic Health Academy, 11405 87th Avenue, Edmonton T6G 1C9, Canada

**Keywords:** Intercultural communication, Cultural competency in nursing, Immigration in Spain, Moroccan immigrants

## Abstract

**Background:**

During the last 25 years, cultural diversity has increased substantially with global migration. In more recent years this has become highly evident in the south of Spain with its steadily increasing Moroccan population. The accompanying differences in ethnocultural values and traditions between the host and newcomer populations may greatly impact healthcare interactions and thus also effective provision of care. This landscape provides for excellent exploration of intercultural communication in healthcare settings and elucidation of possible ways to overcome existing barriers to provision of culturally competent care by nurses. This study aimed to ascertain how nurses perceive their intercultural communication with Moroccan patients and what barriers are evident which may be preventing effective communication and care.

**Methods:**

A focused ethnography was conducted with semi-structured interviews of 32 nurses in three public hospitals in southern Spain. Interviews were audio-recorded and transcribed verbatim before undergoing translation and back-translation between Spanish and English. Data was managed, classified and ordered with the aid of AQUAD.6 (Günter L. Huber, Tübingen, Germany) qualitative data analysis software.

**Results:**

As an important dimension of cultural competence, findings from the interviews with nurses in this study were interpreted within the framework of intercultural communication. Various barriers, for which we have termed “boundaries”, seem to exist preventing effective communication between nurses and their patients. The substantial language barrier seems to negatively affect communication. Relations between the nurses and their Moroccan patients are also marked by prejudices and social stereotypes which likely compromise the provision of culturally appropriate care.

**Conclusions:**

The language barrier may compromise nursing care delivery and could be readily overcome by implementation of professional interpretation within the hospital settings. Moreover, it is essential that the nurses of southern Spain are educated in the provision of culturally appropriate and sensitive care.

## Background

Until the late twentieth century, the ethnocultural profiles of both the nurses and the populations served in hospitals of southern Spain were largely homogenous, being for the most part Spanish citizens of Catholic background. In recent years, ethnocultural diversity has steadily increased through immigration; in fact, since 2000 Spain has seen one of the greatest levels of immigration in the European Union. From 2000 to 2010, the country had the highest increase in migration of any member states, with an increase from 648,533 to 5,650,968 immigrants [1]. The largest ethnocultural group has its origins in Morocco, with almost all immigrants from Morocco being of the Sunni Muslim faith [2,3]. Most recently, in 2010 and 2012 respectively, there were 788,768 and 859,105 Moroccan immigrants with a residence card in Spain [2].

The demographic profile of Moroccan immigrants in Spain shows a clear male dominance (64%) and very different migration profiles by gender [4,5]. Males typically migrate for economic reasons (62%), while women predominantly migrate along with their family (67%). More than half of adult women (53%) have never had a job in Spain, a circumstance which affects only 7% of men who are generally economically active despite their limited opportunities beyond low-skilled and often temporary employment in agriculture and construction. For an important sector of the female population, domestic life has limited their ability to learn Spanish and thus hindered their ability to integrate into various areas of everyday life, such as participating in the child’s education.

Various studies addressing cultural disparity by nationality in Spain have indicated that Moroccans are the foreigners with the greatest cultural distance to Spaniards, primarily due to their Muslim religion [6,7]. There is rejection of the intense religiosity attributed to them by the host community, since it is considered to influence their daily life too much and to hinder their integration into Spanish society. It is very important to realize that individuals who hold Islamic faith are not a homogeneous group, since they are distributed all over the world and represent various national, cultural, political, socioeconomic and ethnic origins. However, there are some common practices throughout the Islamic world because the religion of Islam guides all facets of life, from birth, marriage and family, to politics, economy, and social relations [8,9]. The perceptions and expectations held by Moroccan immigrants regarding the concept of health are primarily shaped by their cultural beliefs and traditions. For example, the Moroccan woman during pregnancy may not attend visits recommended by the hospital largely because of an understanding of medicine as curative rather than preventive [10,11]. Unfortunately, although these belief-led behaviors may result in unaddressed needs or even poor outcomes for some women, there has been a paucity of research mapping out this relationship in the context of Spain’s healthcare system.

When caring for different ethnocultural groups*,* such as the Moroccans within Spain, nurses should ideally have awareness of and appreciation for the specific cultural and religious values and beliefs which may relate to their ability to provide culturally competent care [12-14]. These values often greatly influence human behaviour, providing the basis for decisions and for assessing our own actions against those of others [15]. Beliefs may also shape one’s worldview surrounding life, death and the health-disease process, such as how one falls ill, how to heal, and who can help one recover [16]. Each culture defines or provides a unique set of answers to the health-illness process, considering the condition of the patient, and incorporating persons, interpretations and attitudes that allow for living the disease in a certain way [17].

In the process of achieving culturally competent care provision, nurses should not only know and take into account the degree to which cultural affiliation of a patient determines his/her view of the health-disease process, but they will also have to learn how to communicate effectively with these patents [13,14,18,19]. Indeed, communication is a key variable within several definitions of cultural competence [19-22]. One useful, succinct, definition of cultural competence provided by Fantini [23] is “a complex of abilities needed to perform *effectively* and *appropriately* when interacting with others who are linguistically and culturally different from oneself” (p. 12). Interpreting the findings from interviews with nurses in this study within the framework of intercultural communication may help to determine barriers to provision of cultural competence in this context. Three components of intercultural communication have been described [24]:

• A *cognitive component* encompassing knowledge, understanding and awareness of all the cultural and communicative elements, both in oneself and others, in order to promote effective communication.

• An *emotional component* that includes the capacity for expressing positive emotional responses and to control emotions that may harm the intercultural communication process.

• A *behavioural component* that covers all verbal and non-verbal skills and demonstrates a behavioural adaptation conducive to appropriate and effective communication, in a given context.

The emerging importance of investigating the knowledge and skills favouring intercultural communication in nursing and leading to improved care led us to our study aim of determining how nurses perceive their communication with their Moroccan patients and of identifying relevant barriers that exist for provision of culturally competent care. Apart from identifying themes in our data specific to the voices of the nurses, we also reflected on how our findings could be interpreted within the components of intercultural communication.

## Methods

### Design and setting

To achieve our objective a qualitative methodology was used, since this would provide the best way of entering the level of discourse to attain a deep and rich understanding of the phenomena - an understanding that takes into account the circumstances of the participants and their culture [25]. The study design utilized the principles associated with a focused ethnography and an approach located in the interpretive paradigm. Focused ethnographies are well suited for health research when answering specific questions formulated prior to conducting the research, and are characterized by:

• The focus on a discrete community or organization or social phenomena;

• Being problem-focused and context-specific;

• Involvement of a limited number of participants;

• Participants who usually hold specific knowledge;

• Their use for development in health services [26,27] and

• Episodic (short-term) or no participant observation [28,29].

The study sought to ask nurses to consider their interactions with their Moroccan immigrant patients and to share their perceptions of their communication with their Moroccan patients and to identify relevant barriers that exist when attempting to provide culturally competent care. Other objectives were to identify and understand the beliefs and attitudes held by nurses towards these patients and the knowledge nurses had acquired related to the cultural practices, customs and religions of this patient population.

The study setting consisted of general hospital units (i.e. emergency, intensive care and surgical units were avoided) within three public hospitals in southern Spain, in order to assess our objectives within the context of the close relationship required by the staff with their patients and their families in these settings.

As the research progressed, we became acutely aware that within qualitative research the researcher is regarded as the human instrument [30]. This awareness led the research team to meet during the data analysis stages, with the goal of reflecting on our role and potential influence.

### Sampling and recruitment

Purposive sampling (that is selecting participants with specific characteristics) was undertaken to ensure there was representation of nurses working in various hospital services and who had experience with caring for patients of different ethnocultural groups. The nurses were excluded if they had worked for less than one year providing general care in the hospital of their employment, to help assure fairly extensive exposure to the specific setting. Participation was voluntary and informed consent was obtained from all participants. The participants were recruited by the principal investigator, although the senior nurse manager facilitated the process by helping to locate and contact the nurses who met the criteria for inclusion in the study.

In qualitative research, the number of participants recruited often depends on the saturation of information collected in relation to the research objectives, thus the sample size was not determined prior to study commencement and was determined through an iterative process with on-going data analysis. In total 32 nurses having ethnoculturally homogeneous backgrounds (Spanish heritage and Catholic religion) were recruited, largely consisting of women in the mid-point of their careers. The feminization of the nursing profession and the tendency of male nurses to work in specialized acute care services (such as operating and emergency rooms) resulted in the inclusion of only six male participants.

### Data collection

Data were collected through semi-structured interviews, following an interview guide based on a set of open-ended questions to facilitate in-depth discussion of the topics of interest. The interviews were conducted in a location selected by the informants, which in most cases was the hospital where they worked, in order to establish a suitable environment for both the interviewer and interviewee to facilitate expressions of feelings and emotions in an atmosphere of sincerity. The interviews were audio-taped with the participant’s permission.

### Data management and analysis

**Figure 1:**
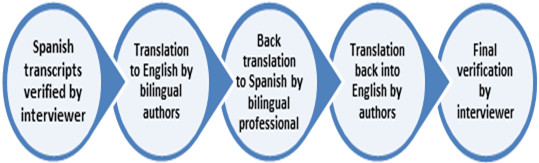
Process of translation and back translation used to ensure transcript accuracy in English.

The Spanish audio-recordings were transcribed verbatim by a professional transcriptionist and verified by the respective interviewer. Subsequently, translation and back-translation was performed to ensure the quality of the English translations. For this, four steps were taken: 1) translation from Spanish to English by the authors of the article, 2) back translation into Spanish by a bilingual professional, 3) translation back into English, and then 4) a final check of the accuracy and quality of the translation by the interviewer (Figure 1). Data was stored, managed, classified and ordered with the aid of AQUAD.6 (Günter L. Huber, Tübingen, Germany) qualitative data analysis software. After transcription, the interviews were reviewed by the interviewer and the interviewee to check the quality of the transcription. *Open coding* of the interview data was performed, whereby we identified themes and key patterns [31] in order to generate the first categories to work with.

### Ethical considerations

Ethical approval of the protocol including recruitment methods was obtained from the Research Subcommittee of Quality, Research and Knowledge Management of Torrecardenas Hospital Complex in Almeria, covering the investigators’ institution and institutions within the governance of the health authority. A code was assigned to each study participant in order to ensure anonymity and confidentiality. In all cases, a written informed consent form was provided.

## Results and discussion

After initially identifying approximately 80 codes in the data, more salient codes emerged to enable grouping of data into key categories. Further analysis allowed identification of four major themes, which we defined in relation to the concept of *border*, “those imaginary lines that are drawn for the boundaries of a country and separating it from one or more neighbouring countries, thus defining the territory in which sovereignty is exercised by everyone” [32]. Correspondingly, the themes of cultural, social, and language boundaries represented imaginary lines separating, pre-disposing and contributing to the nurses’ social positioning in relation to their Moroccan patients. We also present an additional theme relating to *overcoming borders*, as proposed by the informants.

### The cultural boundary

This boundary was used to understand whether or not certain prejudices and stereotypes about Moroccans present a major obstacle in creating a closer personal relationship and cultural approach between nurses and Moroccan patients. A *cultural border* essentially distinguishes between those who are *one of us* or *like us* and others, i.e., *those that come from outside.* One nurse admitted that with the passage of time a mutual understanding,*“*develops during a hospital stay, interpersonal relationships improve” and “if you know them it is another matter, they are no longer fundamentalist, intolerant, filthy” (female, aged 40).

Appearing frequently throughout the interview excerpts was the word “Moor” which has historical connotations. In the year 711, most of the people invading Spain, headed by the Arabs, were from the Berber tribes of North Africa. The tribes of this region, also named Mauritania (from here the word “moor”), were in political alliance with the Umayyad Caliphate of Damascus. They were Islamized and adopted Arabic as their language [33].

The on-going patient contact appeared to result in an overcoming of prejudices and a changing perception that the Moroccan patient is similar to any other patient. However, there seemed to become the growing impression that these patients were more special and unique due to their cultural background, as reported by one of our informants when she described how “they eat with their hands and during mid-afternoon toss a mat on the floor and begin to pray” (female, aged 50). This perception also transferred to patients other than those directly cared for, which was illustrated through many other comments such as “I have not seen a companion treat a *Moor* worse than another patient for simply being a *Moor*” (female, aged 50). In most cases the patients were described as being “very quiet” and neither more troublesome, difficult, or problematic than others.

The prejudices about Moroccans uttered by nurses seemed in most cases to be an *expression of relief*, as we were told sincerely by a male nurse, “but perhaps it is more a perception of comfort that we share among ourselves, the Moor as such…, but I think it is more a thing of habit than contempt” (male, aged 39). The prejudices that exist in the community, and therefore also among nurses, cannot be clearly explained which was witnessed by another informant, “don’t know, I don’t know whether it’s something residing deep within us” (female, aged 40). Moroccans were described as being blamed for problems regarding the respect for and implementation of hospital standards; although the reality is that, when asked about specific problems, conflicts are more or less anecdotal and concern individual patients who were perceived as problematic. Many seemed to consider the Moroccan to be slightly demanding and to define them as *sufferers* resigned to their situation.

### The social boundary

References to xenophobic prejudices among Spanish patients occurred repeatedly in the analyzed data, as we were told by different informants in the hospitals being studied:

Look, I think if there is a problem, not so much with the nursing staff toward them, but rather among patients [such as Spanish patients] who do not want to be with other races, … here we do not care much for the Moors, we do not want them (female, aged 29)

Individual professionals are often sympathetic to these comments and xenophobic expressions, and thereby tend to justify them by making them acceptable. One woman commented “If it is true that other Spanish patients do not want to get involved with Moroccans, simply due to the matter of them being smelly” (aged 52).

Sometimes this sympathy with the xenophobic attitude of Spanish patients appeared to be taken to extremes by the adoption of discriminatory measures, such as allocating patients to rooms according to nationality. This practice was unceremoniously defended as being normal and benefitting Spanish patients, foreign patients and the professional, as witnessed by these the following comment:

Certainly, I put Arabs together with other Arabs, due to the language problem, because, as I say, if I put two together, maybe they don’t understand me, if a family talks, they serve as interpreters (female, aged 29)

This social boundary that society implements may help determine the relationship nurses have with their Moroccan patients, since the indigenous population was described as provoking conflicts from being together with Muslim patients, by for example rejecting to share a room with a Moroccan, or demanding to be attended to ahead of foreign patients.

### The language boundary

The lack of training and thus skills in intercultural care and the need for adapting care to the patient’s culture, seemed to be a primary reason for the problems of cohabitation and the misunderstandings which were too often blamed on the language barrier alone. Informants contributed to this concept on numerous occasions through the following statements:

… the problem is that sometimes there is no one who will translate and there is no good communication, there is a great distance and sometimes I understand nothing (female, aged 38)

… with Moroccan patients, the problems we have is that women do not speak Spanish, so communication with them is very poor (female, aged 43)

… first I need to understand you, at least so we can talk, because if do not understand you, it is very difficult to explain things (female, aged 52)

In such *non-communicative* situations, the staff attempted to resolve the issue by making extra efforts, including the use of gestures or by looking for another patient to act as translator. These efforts were described as allowing enough understanding to enable attention to the patient’s basic needs, such as hunger and hygiene. Literal expressions of this included,

With the Moroccan, it is sign language, gestures, …and if complicated information, I can manage only if we are dealing with simple matters, but with more complicated matters, not even gestures or anything else will work (female, aged 53),

and,

Normally I put in an gigantic effort to get them to speak slowly, using simple words, using a lot of gestures, and this helps, because certain things can be understood by gestures (…) also what we are trying to do is that when we remember that there is another of the same nationality in another room that speaks our language a little more he/she may help us to translate (female, aged 49)

Nurses may not be aware of the contribution of cultural differences to misunderstandings, as we were told by one nurse, “a patient, whom we believed refused to follow a treatment, but in reality after a translation, we understood why, and we came to a mutual decision and solved the problem" (female, aged 32).

### Overcoming borders

Several nurses provided solutions to overcome the *borders* that separate them from their *Moroccan* patients, largely as related to improving communication. For many, all *borders* are reduced to one, the language barrier, which is expressed below by two informants as a great need for interpreters:

… language, this applies to both Arabs and other foreigners, is what matters, they need to improve their language skills or a person or an interpreter must be present in order to translate for us, many times, you don’t know whether treatments are being followed or if they do what you tell them, either because they don’t want to or because they do not understand (female, aged 40)

Therefore, I say to you that an interpreter would be ideal, at least so that they can tell you what’s going on (female, aged 37)

It should be emphasized that the study participants did not know about, or at least did not mention, the role of an intercultural mediator; only one person referred to what such a professional might be: “there were persons trained in this religion and its affairs, and who provide courses” (female, aged 37). Some professionals do sense the need for intercultural education, but are unable to nail down such a shortcoming in concrete terms, as expressed in the following excerpts of two interviews:

I think it is the ignorance not knowing their culture, we do things our way, regardless of whether they like it or not, or is beneficial for them, we just go ahead and serve a pork-free diet (female, aged 40)

… know their habits a bit more and a little bit about their experiences with their disease, having more information available (female, aged 41)

In other cases it comes down to appreciating the need for finding a cultural approach, even though it calls for an “adjustment by both parties”, as one nurse informed us.

Having more communication will make us more accommodating toward them, and better appreciate what is happening. There should be flexibility on either side, i.e. us trying to understand their situation being in a foreign country and not understanding you, I believe it gives rise to great anxiety finding yourself in a place where you are unable to explain your feelings, and similarly, they should adjust to the norms of the institution. That plus a little adjustment on our part, as well as theirs, doing both our shares so that care may be improved (female, aged 53)

On the topic of hospital management, nurses often voiced their criticism, as reflected below:

From the management, we need training on how immigrants experience health and illness, to know them a little better culturally (female, aged 37)

This is totally unrealistic (laughs) … how are we going to get a translator in here, that’s impossible (female, aged 43)

… they are asleep at the wheel, know nothing, won’t respond, of course many things can be done, for example, as far as I recall they have not provided any continuing education courses in such very basic matter as Moroccans’ customs in general, which would have been a good idea… they could do many things, but they never do (female, aged 49)

### Limitations of the study

We appreciate that this study is limited by the study population, itself, in two ways. The study relates strictly to hospital nurses; our findings are thus a reflection of hospital nurses’ approaches to situations and their personal experiences. Secondly, it would be of great interest to explore the perceptions of the nurse-patient relationship from the vantage point of our study population, as users of the public health system and a source of comparison. Another study has commenced which will help provide this understanding.

## Conclusions

In southern Spain, caring for immigrant patients has taken many nurses and health institutions by surprise, and for many there has been no education or special training provided. The increasing number of immigrants entering Europe will likely continue unabated, making the cultural profile of health care users increasingly diverse. This is particularly noticeable in southern Spain where if the migration trend of the Moroccan community continues, their presence in Spain will be permanent and increase progressively. Consequently, intercultural encounters in hospitals within south Spain have gone from being an exception to a daily occurrence. This study demonstrated that the language barrier during communication with Moroccan patients is real and commonplace yet could be easily resolved by hiring translators at hospitals.

However, it is our conclusion that the views, opinions, and attitudes of nurses, like the rest of society, are shaped by prejudices and stereotypes that have roots deep in Western culture whose common denominator is the rejection of the Moor*.* The Spanish have a relationship of power with migrants arriving from Morocco, but are unaware that they manifest these attitudes. It is a relationship between the East and the West, in which the East is subordinated to the West and the West emits the collective notion that defines the “we” who are the Europeans against the “strange” that is “other” [34]. It is an opposition between two worlds, two cultures, and two styles. Western people have a subtle and persistent Eurocentric prejudice against Arab or Islamic peoples and their culture, and it is manifested in the relationship that the host has with the immigrant.

One major difference between nurses and the rest of society is that they have an opportunity, although not initially chosen or desired, to shed these preconceptions through their personal and professional contact with these patients. The ensuing intercultural relationship permits a contrast of these prejudices with reality, whereby awareness and understanding can form. Nurses could become important players in challenging the stereotypes and prejudices held by Spanish patients about immigrants. The notable absence of conflicts with Moroccan patients, and the apparent susceptibility of nurses to cultural awareness and sensitivity, allows for some optimism for the future.

Cultural competence would theoretically improve patient outcomes and satisfaction, since many ethnocultural groups value relational quality and thus highly interpersonal care. Unfortunately, to date there is a paucity of primary research that specifically investigates the use of a model or tool in improving nursing care delivery and patient satisfaction/outcomes [35]. In general, most of the evidence related to models and tools developed to enhance cultural competency in nursing practice has focused primarily on education and training of nurses and health professionals. Largely within social science but also some medical fields such as mental health, there are other theoretical and conceptual frameworks which are summarized by Collins and Guruge [36]. While these frameworks have not tended to underpin specific care assessment or delivery tools used in clinical nursing practice, they may assist nurses and health care practitioners in understanding the social positioning of the diverse ethnocultural groups for whom they endeavour to deliver high quality care. Models of cultural competency for practice and training specific to the Spanish context of this study are needed.

### Recommendations for the training of nurses

We propose a commitment to training nurses to overcome prejudices and stereotypes when attending to and caring for the universality of patients. To improve social harmony, cultural respect and quality of care, nurses must master intercultural communication and their ability to appropriately adapt their nursing care to various ethnocultural groups. Part of this process will depend on their becoming knowledgeable about other cultures and alternative ways of experiencing the health-disease process, but more importantly about nursing theories and models exploring cultural care and cultural nursing competence [13,14,35,37].

## Competing interests

The authors declare that they have no competing interests.

## Authors’ contributions

Both FP and ES participated in every stage of the study. FP and ES were responsible for the study conception and the design and drafting of the manuscript. FP and ES performed the data collection and the data analysis. GH assisted with developing the study design and critically edited the manuscript. All authors read and approved the final manuscript.

## Authors’ information

Gina Higginbottom: http://www.chairs-chaires.gc.ca/.

## Pre-publication history

The pre-publication history for this paper can be accessed here:

http://www.biomedcentral.com/1472-6955/12/14/prepub
